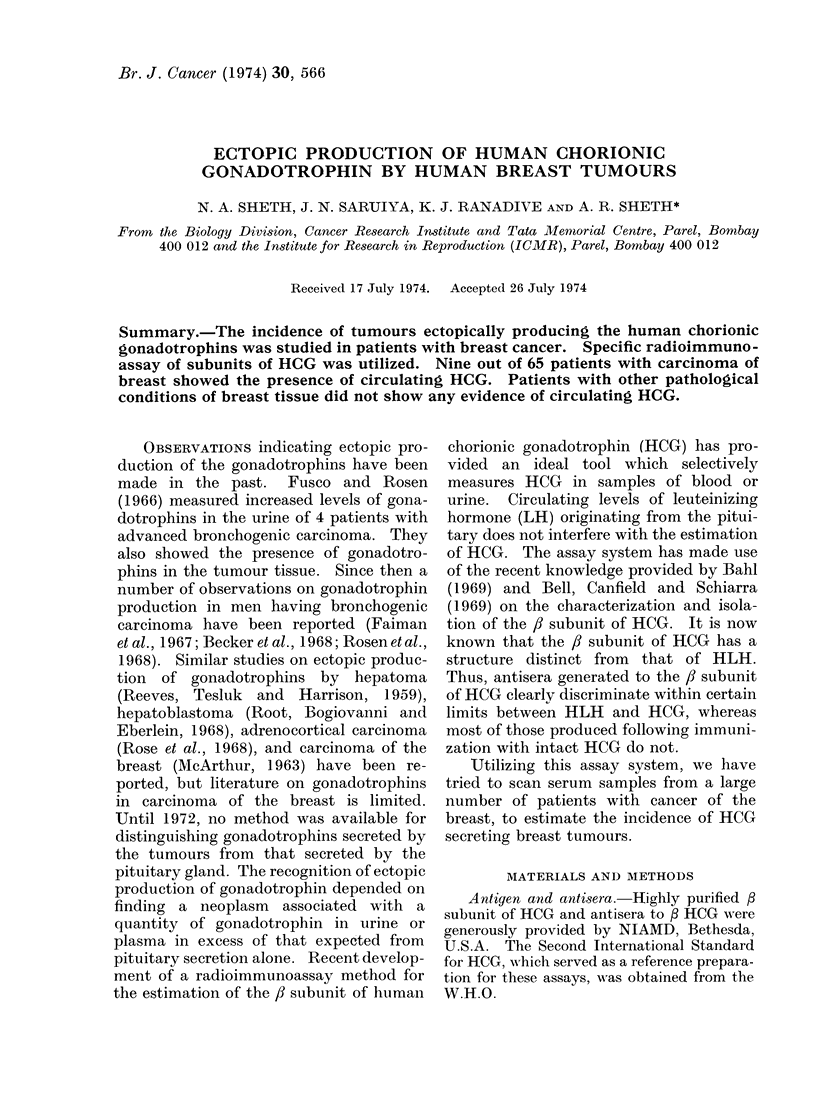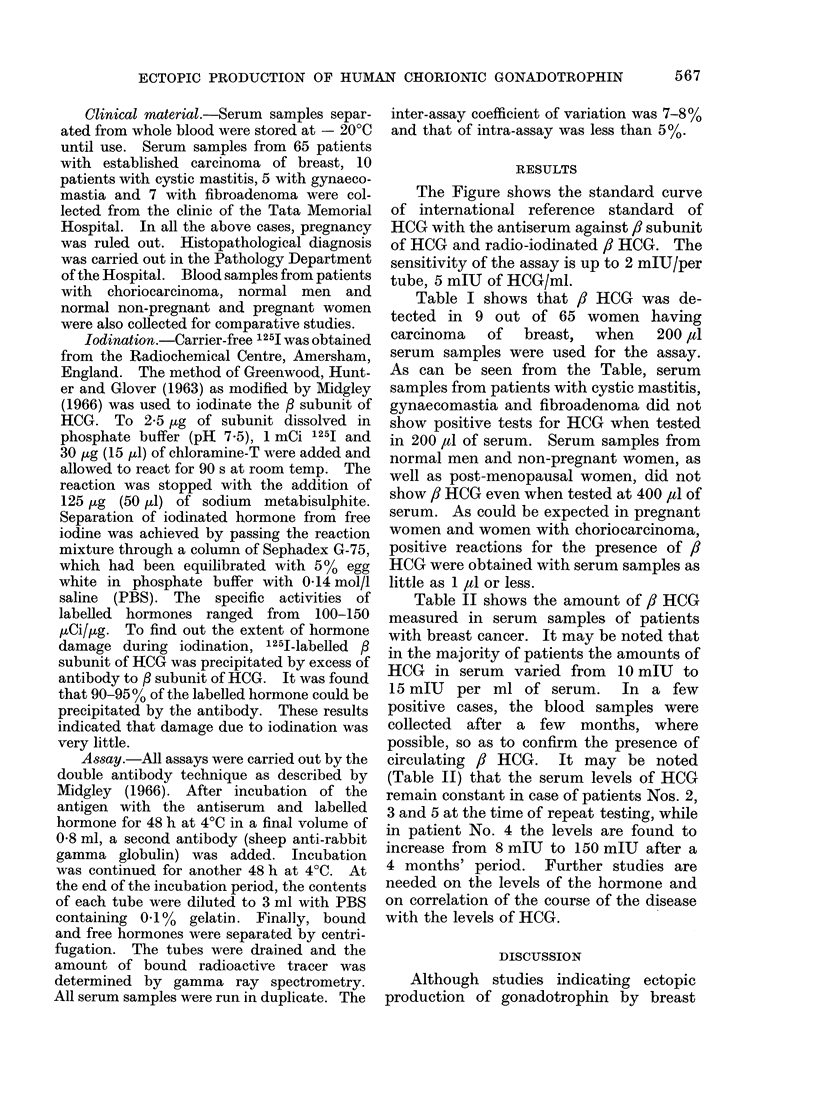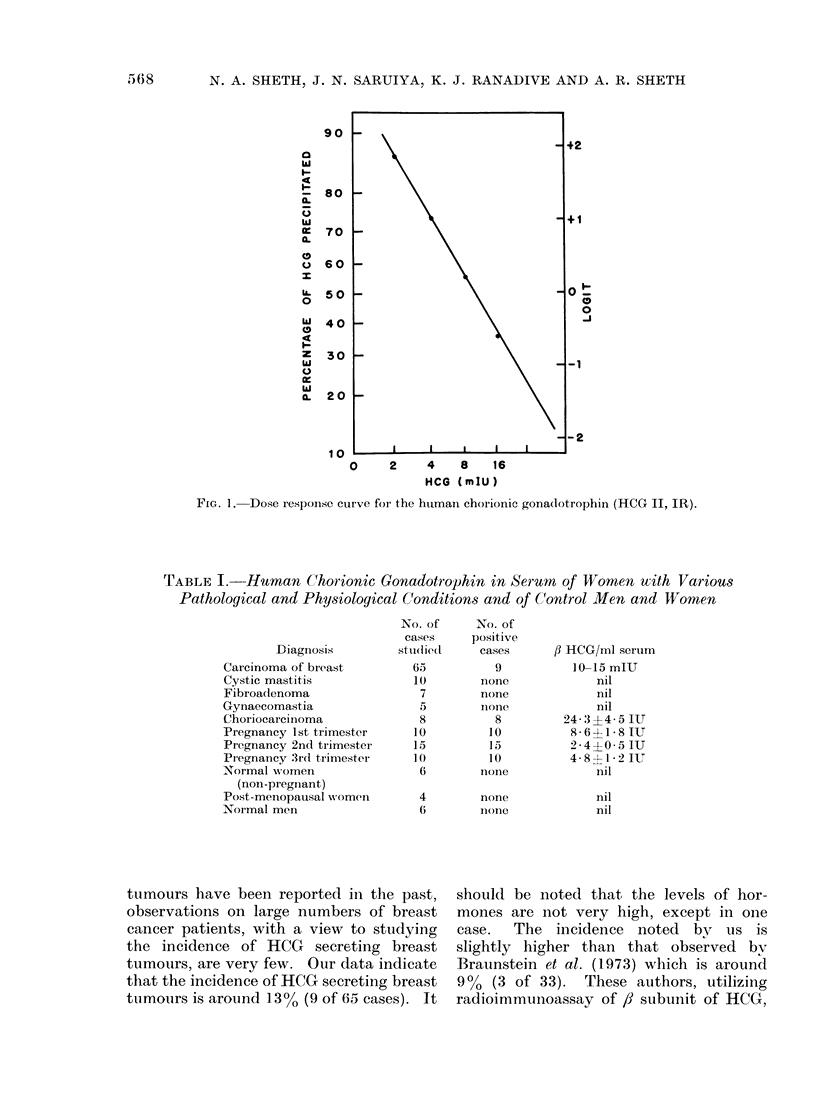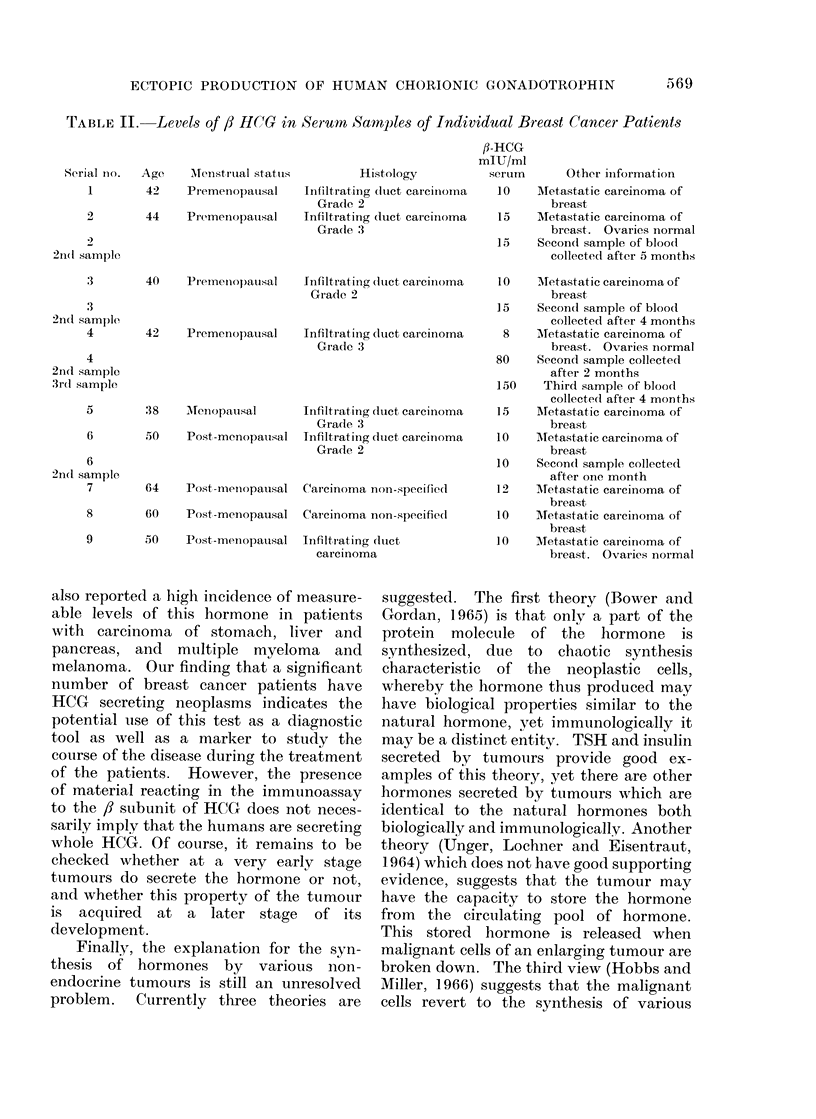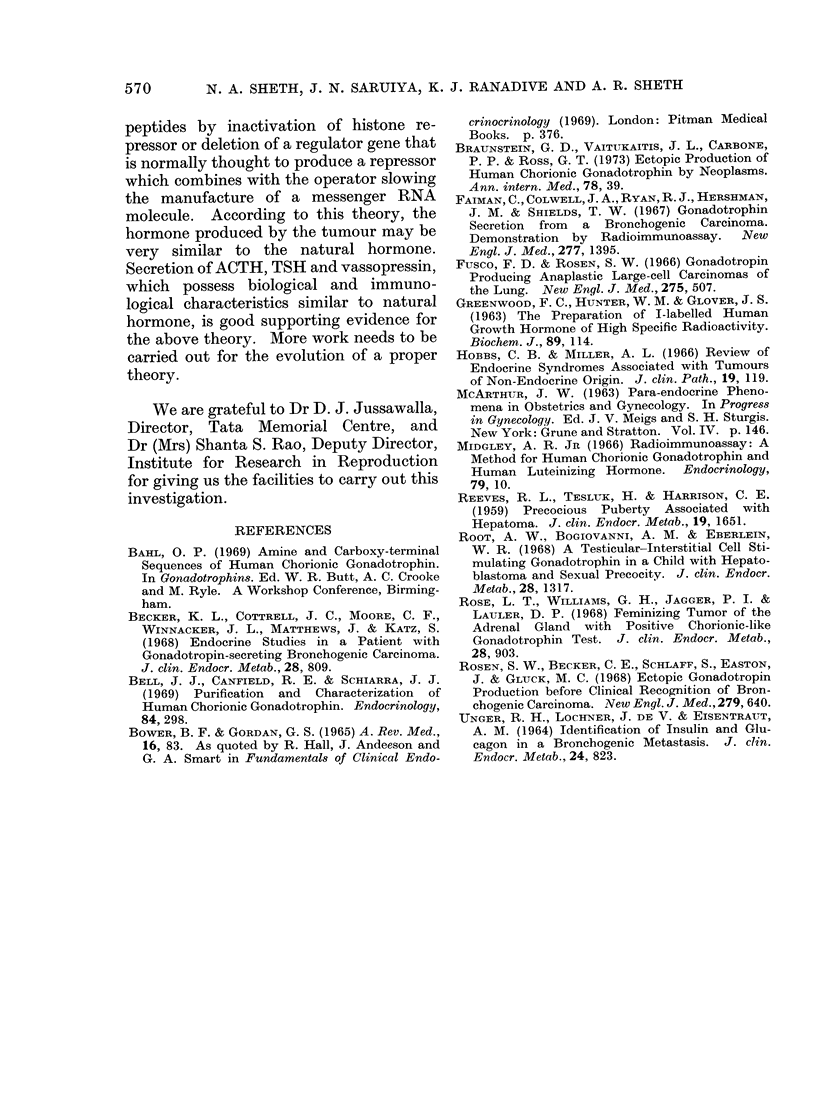# Ectopic Production of Human Chorionic Gonadotrophin by Human Breast Tumours

**DOI:** 10.1038/bjc.1974.236

**Published:** 1974-12

**Authors:** N. A. Sheth, J. N. Saruiya, K. J. Ranadive, A. R. Sheth

## Abstract

The incidence of tumours ectopically producing the human chorionic gonadotrophins was studied in patients with breast cancer. Specific radioimmunoassay of subunits of HCG was utilized. Nine out of 65 patients with carcinoma of breast showed the presence of circulating HCG. Patients with other pathological conditions of breast tissue did not show any evidence of circulating HCG.


*Br. J. Cancer* (1974) **30**, 566

### ECTOPIC PRODUCTION OF HUMAN CHORIONIC GONADOTROPHIN BY HUMAN BREAST TUMOURS

N. A. SHETH, J. N. SARUIYA, K. J. RANADIVE AND A. R. SHETH*

*From the Biology Division, Cancer Research Institute and Tata Memorial Centre, Parel, Bombay* 400 012 *and the Institute for Research in Reproduction (ICMR), Parel, Bombay* 400 012

Received 17 July 1974. Accepted 26 July 1974

**Summary.—The incidence of tumours ectopically producing the human chorionic gonadotrophins was studied in patients with breast cancer. Specific radioimmunoassay of subunits of HCG was utilized. Nine out of 65 patients with carcinoma of breast showed the presence of circulating HCG. Patients with other pathological conditions of breast tissue did not show any evidence of circulating HCG.**

OBSERVATIONS indicating ectopic production of the gonadotrophins have been made in the past. Fusco and Rosen (1966) measured increased levels of gonadotrophins in the urine of 4 patients with advanced bronchogenic carcinoma. They also showed the presence of gonadotrophins in the tumour tissue. Since then a number of observations on gonadotrophin production in men having bronchogenic carcinoma have been reported (Faiman *et al.*, 1967; Becker *et al.*, 1968; Rosen *et al.*, 1968). Similar studies on ectopic production of gonadotrophins by hepatoma (Reeves, Tesluk and Harrison, 1959), hepatoblastoma (Root, Bogiovanni and Eberlein, 1968), adrenocortical carcinoma (Rose *et al.*, 1968), and carcinoma of the breast (McArthur, 1963) have been reported, but literature on gonadotrophins in carcinoma of the breast is limited. Until 1972, no method was available for distinguishing gonadotrophins secreted by the tumours from that secreted by the pituitary gland. The recognition of ectopic production of gonadotrophin depended on finding a neoplasm associated with a quantity of gonadotrophin in urine or plasma in excess of that expected from pituitary secretion alone. Recent development of a radioimmunoassay method for the estimation of the $\beta$ subunit of human chorionic gonadotrophin (HCG) has provided an ideal tool which selectively measures HCG in samples of blood or urine. Circulating levels of leuteinizing hormone (LH) originating from the pituitary does not interfere with the estimation of HCG. The assay system has made use of the recent knowledge provided by Bahl (1969) and Bell, Canfield and Schiarra (1969) on the characterization and isolation of the $\beta$ subunit of HCG. It is now known that the $\beta$ subunit of HCG has a structure distinct from that of HLH. Thus, antisera generated to the $\beta$ subunit of HCG clearly discriminate within certain limits between HLH and HCG, whereas most of those produced following immunization with intact HCG do not.

Utilizing this assay system, we have tried to scan serum samples from a large number of patients with cancer of the breast, to estimate the incidence of HCG secreting breast tumours.

### MATERIALS AND METHODS

*Antigen and antisera.*—Highly purified $\beta$ subunit of HCG and antisera to $\beta$ HCG were generously provided by NIAMD, Bethesda, U.S.A. The Second International Standard for HCG, which served as a reference preparation for these assays, was obtained from the W.H.O.

*Clinical material.*—Serum samples separated from whole blood were stored at − 20°C until use. Serum samples from 65 patients with established carcinoma of breast, 10 patients with cystic mastitis, 5 with gynaecomastia and 7 with fibroadenoma were collected from the clinic of the Tata Memorial Hospital. In all the above cases, pregnancy was ruled out. Histopathological diagnosis was carried out in the Pathology Department of the Hospital. Blood samples from patients with choriocarcinoma, normal men and normal non-pregnant and pregnant women were also collected for comparative studies.

*Iodination.*—Carrier-free $^{125}I$ was obtained from the Radiochemical Centre, Amersham, England. The method of Greenwood, Hunter and Glover (1963) as modified by Midgley (1966) was used to iodinate the $\beta$ subunit of HCG. To 2·5 $\mu$g of subunit dissolved in phosphate buffer (pH 7·5), 1 mCi $^{125}I$ and 30 $\mu$g (15 $\mu$l) of chloramine-T were added and allowed to react for 90 s at room temp. The reaction was stopped with the addition of 125 $\mu$g (50 $\mu$l) of sodium metabisulphite. Separation of iodinated hormone from free iodine was achieved by passing the reaction mixture through a column of Sephadex G-75, which had been equilibrated with 5% egg white in phosphate buffer with 0·14 mol/l saline (PBS). The specific activities of labelled hormones ranged from 100–150 $\mu$Ci/$\mu$g. To find out the extent of hormone damage during iodination, $^{125}I$-labelled $\beta$ subunit of HCG was precipitated by excess of antibody to $\beta$ subunit of HCG. It was found that 90–95% of the labelled hormone could be precipitated by the antibody. These results indicated that damage due to iodination was very little.

*Assay.*—All assays were carried out by the double antibody technique as described by Midgley (1966). After incubation of the antigen with the antiserum and labelled hormone for 48 h at 4°C in a final volume of 0·8 ml, a second antibody (sheep anti-rabbit gamma globulin) was added. Incubation was continued for another 48 h at 4°C. At the end of the incubation period, the contents of each tube were diluted to 3 ml with PBS containing 0·1% gelatin. Finally, bound and free hormones were separated by centrifugation. The tubes were drained and the amount of bound radioactive tracer was determined by gamma ray spectrometry. All serum samples were run in duplicate. The inter-assay coefficient of variation was 7–8% and that of intra-assay was less than 5%.

### RESULTS

The Figure shows the standard curve of international reference standard of HCG with the antiserum against $\beta$ subunit of HCG and radio-iodinated $\beta$ HCG. The sensitivity of the assay is up to 2 mIU/per tube, 5 mIU of HCG/ml.

Table I shows that $\beta$ HCG was detected in 9 out of 65 women having carcinoma of breast, when 200 $\mu$l serum samples were used for the assay. As can be seen from the Table, serum samples from patients with cystic mastitis, gynaecomastia and fibroadenoma did not show positive tests for HCG when tested in 200 $\mu$l of serum. Serum samples from normal men and non-pregnant women, as well as post-menopausal women, did not show $\beta$ HCG even when tested at 400 $\mu$l of serum. As could be expected in pregnant women and women with choriocarcinoma, positive reactions for the presence of $\beta$ HCG were obtained with serum samples as little as 1 $\mu$l or less.

Table II shows the amount of $\beta$ HCG measured in serum samples of patients with breast cancer. It may be noted that in the majority of patients the amounts of HCG in serum varied from 10 mIU to 15 mIU per ml of serum. In a few positive cases, the blood samples were collected after a few months, where possible, so as to confirm the presence of circulating $\beta$ HCG. It may be noted (Table II) that the serum levels of HCG remain constant in case of patients Nos. 2, 3 and 5 at the time of repeat testing, while in patient No. 4 the levels are found to increase from 8 mIU to 150 mIU after a 4 months' period. Further studies are needed on the levels of the hormone and on correlation of the course of the disease with the levels of HCG.

### DISCUSSION

Although studies indicating ectopic production of gonadotrophin by breast

FIG. 1.—Dose response curve for the human chorionic gonadotrophin (HCG II, IR).

TABLE I.—*Human Chorionic Gonadotrophin in Serum of Women with Various Pathological and Physiological Conditions and of Control Men and Women*

| Diagnosis | No. of cases studied | No. of positive cases | β HCG/ml serum |
|---|---|---|---|
| Carcinoma of breast | 65 | 9 | 10–15 mIU |
| Cystic mastitis | 10 | none | nil |
| Fibroadenoma | 7 | none | nil |
| Gynaecomastia | 5 | none | nil |
| Choriocarcinoma | 8 | 8 | 24·3±4·5 IU |
| Pregnancy 1st trimester | 10 | 10 | 8·6±1·8 IU |
| Pregnancy 2nd trimester | 15 | 15 | 2·4±0·5 IU |
| Pregnancy 3rd trimester | 10 | 10 | 4·8±1·2 IU |
| Normal women (non-pregnant) | 6 | none | nil |
| Post-menopausal women | 4 | none | nil |
| Normal men | 6 | none | nil |

tumours have been reported in the past, observations on large numbers of breast cancer patients, with a view to studying the incidence of HCG secreting breast tumours, are very few. Our data indicate that the incidence of HCG secreting breast tumours is around 13% (9 of 65 cases). It should be noted that the levels of hormones are not very high, except in one case. The incidence noted by us is slightly higher than that observed by Braunstein *et al.* (1973) which is around 9% (3 of 33). These authors, utilizing radioimmunoassay of β subunit of HCG,

TABLE II.—*Levels of β HCG in Serum Samples of Individual Breast Cancer Patients*

| Serial no. | Age | Menstrual status | Histology | β-HCG mIU/ml serum | Other information |
|---|---|---|---|---|---|
| 1 | 42 | Premenopausal | Infiltrating duct carcinoma Grade 2 | 10 | Metastatic carcinoma of breast |
| 2 | 44 | Premenopausal | Infiltrating duct carcinoma Grade 3 | 15 | Metastatic carcinoma of breast. Ovaries normal |
| 2 2nd sample | | | | 15 | Second sample of blood collected after 5 months |
| 3 | 40 | Premenopausal | Infiltrating duct carcinoma Grade 2 | 10 | Metastatic carcinoma of breast |
| 3 2nd sample | | | | 15 | Second sample of blood collected after 4 months |
| 4 | 42 | Premenopausal | Infiltrating duct carcinoma Grade 3 | 8 | Metastatic carcinoma of breast. Ovaries normal |
| 4 2nd sample | | | | 80 | Second sample collected after 2 months |
| 3rd sample | | | | 150 | Third sample of blood collected after 4 months |
| 5 | 38 | Menopausal | Infiltrating duct carcinoma Grade 3 | 15 | Metastatic carcinoma of breast |
| 6 | 50 | Post-menopausal | Infiltrating duct carcinoma Grade 2 | 10 | Metastatic carcinoma of breast |
| 6 2nd sample | | | | 10 | Second sample collected after one month |
| 7 | 64 | Post-menopausal | Carcinoma non-specified | 12 | Metastatic carcinoma of breast |
| 8 | 60 | Post-menopausal | Carcinoma non-specified | 10 | Metastatic carcinoma of breast |
| 9 | 50 | Post-menopausal | Infiltrating duct carcinoma | 10 | Metastatic carcinoma of breast. Ovaries normal |

also reported a high incidence of measureable levels of this hormone in patients with carcinoma of stomach, liver and pancreas, and multiple myeloma and melanoma. Our finding that a significant number of breast cancer patients have HCG secreting neoplasms indicates the potential use of this test as a diagnostic tool as well as a marker to study the course of the disease during the treatment of the patients. However, the presence of material reacting in the immunoassay to the β subunit of HCG does not necessarily imply that the humans are secreting whole HCG. Of course, it remains to be checked whether at a very early stage tumours do secrete the hormone or not, and whether this property of the tumour is acquired at a later stage of its development.

Finally, the explanation for the synthesis of hormones by various non-endocrine tumours is still an unresolved problem. Currently three theories are suggested. The first theory (Bower and Gordan, 1965) is that only a part of the protein molecule of the hormone is synthesized, due to chaotic synthesis characteristic of the neoplastic cells, whereby the hormone thus produced may have biological properties similar to the natural hormone, yet immunologically it may be a distinct entity. TSH and insulin secreted by tumours provide good examples of this theory, yet there are other hormones secreted by tumours which are identical to the natural hormones both biologically and immunologically. Another theory (Unger, Lochner and Eisentraut, 1964) which does not have good supporting evidence, suggests that the tumour may have the capacity to store the hormone from the circulating pool of hormone. This stored hormone is released when malignant cells of an enlarging tumour are broken down. The third view (Hobbs and Miller, 1966) suggests that the malignant cells revert to the synthesis of various peptides by inactivation of histone repressor or deletion of a regulator gene that is normally thought to produce a repressor which combines with the operator slowing the manufacture of a messenger RNA molecule. According to this theory, the hormone produced by the tumour may be very similar to the natural hormone. Secretion of ACTH, TSH and vassopressin, which possess biological and immunological characteristics similar to natural hormone, is good supporting evidence for the above theory. More work needs to be carried out for the evolution of a proper theory.

We are grateful to Dr D. J. Jussawalla, Director, Tata Memorial Centre, and Dr (Mrs) Shanta S. Rao, Deputy Director, Institute for Research in Reproduction for giving us the facilities to carry out this investigation.

### REFERENCES

BAHL, O. P. (1969) Amine and Carboxy-terminal Sequences of Human Chorionic Gonadotrophin. In *Gonadotrophins*. Ed. W. R. Butt, A. C. Crooke and M. Ryle. A Workshop Conference, Birmingham.

BECKER, K. L., COTTRELL, J. C., MOORE, C. F., WINNACKER, J. L., MATTHEWS, J. & KATZ, S. (1968) Endocrine Studies in a Patient with Gonadotropin-secreting Bronchogenic Carcinoma. *J. clin. Endocr. Metab.*, **28**, 809.

BELL, J. J., CANFIELD, R. E. & SCHIARRA, J. J. (1969) Purification and Characterization of Human Chorionic Gonadotrophin. *Endocrinology*, **84**, 298.

BOWER, B. F. & GORDAN, G. S. (1965) *A. Rev. Med.*, **16**, 83. As quoted by R. Hall, J. Andeeson and G. A. Smart in *Fundamentals of Clinical Endocrinocrinology* (1969). London: Pitman Medical Books. p. 376.

BRAUNSTEIN, G. D., VAITUKAITIS, J. L., CARBONE, P. P. & ROSS, G. T. (1973) Ectopic Production of Human Chorionic Gonadotrophin by Neoplasms. *Ann. intern. Med.*, **78**, 39.

FAIMAN, C., COLWELL, J. A., RYAN, R. J., HERSHMAN, J. M. & SHIELDS, T. W. (1967) Gonadotrophin Secretion from a Bronchogenic Carcinoma. Demonstration by Radioimmunoassay. *New Engl. J. Med.*, **277**, 1395.

FUSCO, F. D. & ROSEN, S. W. (1966) Gonadotropin Producing Anaplastic Large-cell Carcinomas of the Lung. *New Engl. J. Med.*, **275**, 507.

GREENWOOD, F. C., HUNTER, W. M. & GLOVER, J. S. (1963) The Preparation of I-labelled Human Growth Hormone of High Specific Radioactivity. *Biochem. J.*, **89**, 114.

HOBBS, C. B. & MILLER, A. L. (1966) Review of Endocrine Syndromes Associated with Tumours of Non-Endocrine Origin. *J. clin. Path.*, **19**, 119.

MCARTHUR, J. W. (1963) Para-endocrine Phenomena in Obstetrics and Gynecology. In *Progress in Gynecology*. Ed. J. V. Meigs and S. H. Sturgis. New York: Grune and Stratton. Vol. IV. p. 146.

MIDGLEY, A. R. JR (1966) Radioimmunoassay: A Method for Human Chorionic Gonadotrophin and Human Luteinizing Hormone. *Endocrinology*, **79**, 10.

REEVES, R. L., TESLUK, H. & HARRISON, C. E. (1959) Precocious Puberty Associated with Hepatoma. *J. clin. Endocr. Metab.*, **19**, 1651.

ROOT, A. W., BOGIOVANNI, A. M. & EBERLEIN, W. R. (1968) A Testicular–Interstitial Cell Stimulating Gonadotrophin in a Child with Hepatoblastoma and Sexual Precocity. *J. clin. Endocr. Metab.*, **28**, 1317.

ROSE, L. T., WILLIAMS, G. H., JAGGER, P. I. & LAULER, D. P. (1968) Feminizing Tumor of the Adrenal Gland with Positive Chorionic-like Gonadotrophin Test. *J. clin. Endocr. Metab.*, **28**, 903.

ROSEN, S. W., BECKER, C. E., SCHLAFF, S., EASTON, J. & GLUCK, M. C. (1968) Ectopic Gonadotropin Production before Clinical Recognition of Bronchogenic Carcinoma. *New Engl. J. Med.*, **279**, 640.

UNGER, R. H., LOCHNER, J. DE V. & EISENTRAUT, A. M. (1964) Identification of Insulin and Glucagon in a Bronchogenic Metastasis. *J. clin. Endocr. Metab.*, **24**, 823.